# Surface-Density-Controlled Spreading–Packing Competition in Antibody Monolayers Revealed by PM-IRRAS and 2D Correlation Spectroscopy

**DOI:** 10.1021/acsomega.6c02134

**Published:** 2026-06-17

**Authors:** Matteo Piscitelli, Cinzia Di Franco, Lucia Sarcina, Michele Catacchio, Fabrizio Corvino, Eleonora Macchia, Luisa Torsi, Gaetano Scamarcio

**Affiliations:** † Dipartimento Interateneo di Fisica, 9295 Università degli Studi di Bari Aldo Moro, Bari 70125, Italy; ‡ Consiglio Nazionale delle Ricerche−Istituto di Fotonica e Nanotecnologie (CNR-IFN), Bari 70125, Italy; § Dipartimento di Chimica, Università degli Studi di Bari Aldo Moro, Bari 70125, Italy; ∥ Dipartimento di Farmacia-Scienze del Farmaco, Università degli Studi di Bari Aldo Moro, Bari 70125, Italy; ⊥ Consiglio Nazionale delle Ricerche−Istituto Nanoscienze (CNR-Nano), Pisa 56127, Italy

## Abstract

Antibody adsorption onto solid surfaces underpins numerous biosensing and bioelectronic platforms, yet quantitative descriptors linking adsorption conditions to interfacial structure remain limited. Here, we quantify the structural evolution of anti-immunoglobulin M (anti-IgM) monolayers physisorbed on a gold substrate. Our investigation spans a wide range of solution concentrations (0.5 to 1000 μg mL^–1^) and deposition times (1 to 330 min), encompassing both dilute and densely packed regimes. Polarization-modulation infrared reflection–absorption spectroscopy (PM-IRRAS) quantifies secondary-structure fractions. Concurrently, asynchronous two-dimensional (2D) correlation spectroscopy resolves the sequential order of conformational changes. A strong anticorrelation between β-sheet and unordered chains content emerges: at low surface density, adsorbed antibodies adopt conformationally flexible, partially disordered states, whereas increasing concentration and incubation time drive compact, β-sheet-rich assemblies. To unify concentration- and time-dependent effects, we introduce a normalized surface-density (SD) parameter derived from PM-IRRAS intensity, which collapses adsorption pathways onto a single structural coordinate. Expressing secondary-structure fractions as a function of SD quantitatively maps the spreading–packing competition governing antibody monolayers. This surface-density-based framework provides a predictive descriptor for adsorption-induced structural heterogeneity in antibody interfaces.

## Graphical abstract

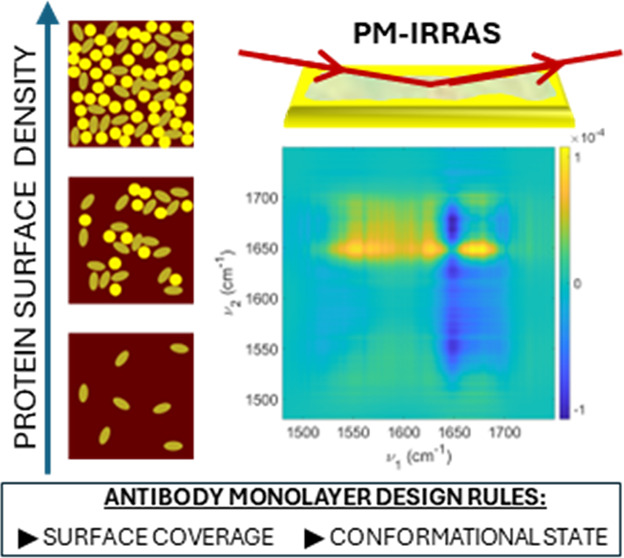

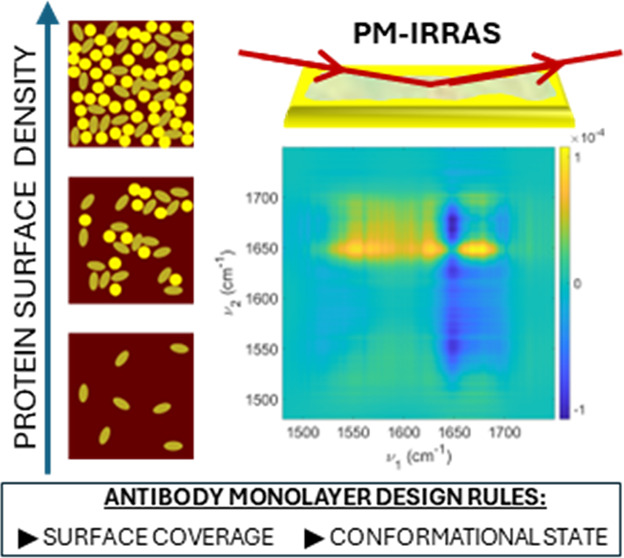

## 1 Introduction

Protein physisorption at solid–liquid interfaces underpins a broad spectrum of biofunctional technologies, from biosensors and lab-on-chip devices to medical implants and drug-delivery platforms. Compared with covalent immobilization, physisorption provides a rapid, low-cost, and scalable route for protein attachment, avoiding complex surface functionalization protocols.^1,2^ However, this spontaneous process is highly sensitive to experimental parameters such as concentration, incubation time, and solvent composition, which can critically affect the structural and functional integrity of adsorbed proteins.

Protein physisorption reflects a subtle balance among protein–surface, protein–protein, and protein–solvent interactions, mediated by van der Waals forces, hydrogen bonding, hydrophobic contacts, and electrostatics. Typically, interfacial interactions promote spontaneous self-assembly into monolayers that minimize interfacial free energy but can perturb the native fold.^3,4^ The adsorption is intrinsically self-limiting: steric hindrance and orientational constraints prevent multilayer buildup, leading to monomolecular films with a thickness corresponding to a single protein layer.^5^ External factors such as pH, ionic strength, temperature, or geometric confinement further modulate this equilibrium, driving adaptive conformational responses. These adaptations may include partial unfolding, rearrangement of secondary motifs, or aggregation.^6,7^

Understanding how proteins behave upon physisorption remains a central challenge in interfacial science. In particular, the adsorption of large and soft proteins with low internal stability, such as antibodies or albumins, is typically described by a random process in which each protein undergoes a two-stage transformation: an initial *spreading* phase, in which individual molecules maximize surface interaction by partial unfolding, followed by a *packing* phase, where crowding and intermolecular interactions constrain molecular motion. Cooperative transitions between extended and compact conformations, driven by the interplay between protein–surface and protein–protein interactions, have been observed in several protein systems, providing experimental and theoretical support for this spreading–packing competition.^8–11^ The interplay between these competing processes dictates both the surface coverage and the conformational state of the adsorbed proteins.

A major difficulty stems from the intrinsic structural heterogeneity of the protein films. Upon adsorption, proteins do not adopt a single well-defined conformation but instead populate a distribution of native-like, partially unfolded, or misfolded states, whose relative abundance depends on the adsorption pathway rather than solely on the final surface coverage.^9^ This heterogeneity is further compounded by ensemble averaging: experimental observables reflect the superposition of signals from molecules in different states, obscuring how adsorption conditions map to specific conformational outcomes. Capturing and quantifying this distribution are therefore essential for understanding how antibodies organize at interfaces and for establishing predictive principles to guide the engineering of functional protein monolayers. While adsorption-induced conformational changes have been extensively documented, quantitative mapping of how adsorption pathways translate to specific structural states remains limited. Most studies independently examine concentration or time effects, and structural interpretations are often qualitative or are inferred indirectly. Moreover, the sequential relationship between molecular reorientation and secondary structure reorganization during adsorption remains insufficiently resolved. A unified descriptor that links adsorption conditions to structural outcomes has not yet been developed.

Polarization-modulation infrared reflection–absorption spectroscopy (PM-IRRAS) is one of the few techniques capable of directly probing nanometer-thin molecular films on metallic substrates. It provides nondestructive, surface-sensitive access to the secondary structure of adsorbed proteins by resolving the amide I and amide II vibrational bands, enabling quantitative assessment of β-sheet, α-helix, and unordered chains content and average orientation.^12,13^ When combined with two-dimensional (2D) correlation analysis, PM-IRRAS reveals the sequential evolution of spectral contributions under external perturbations, offering a dynamic view of adsorption-induced structural transitions^14,15^ (see Figure 1).

**Figure 1:**
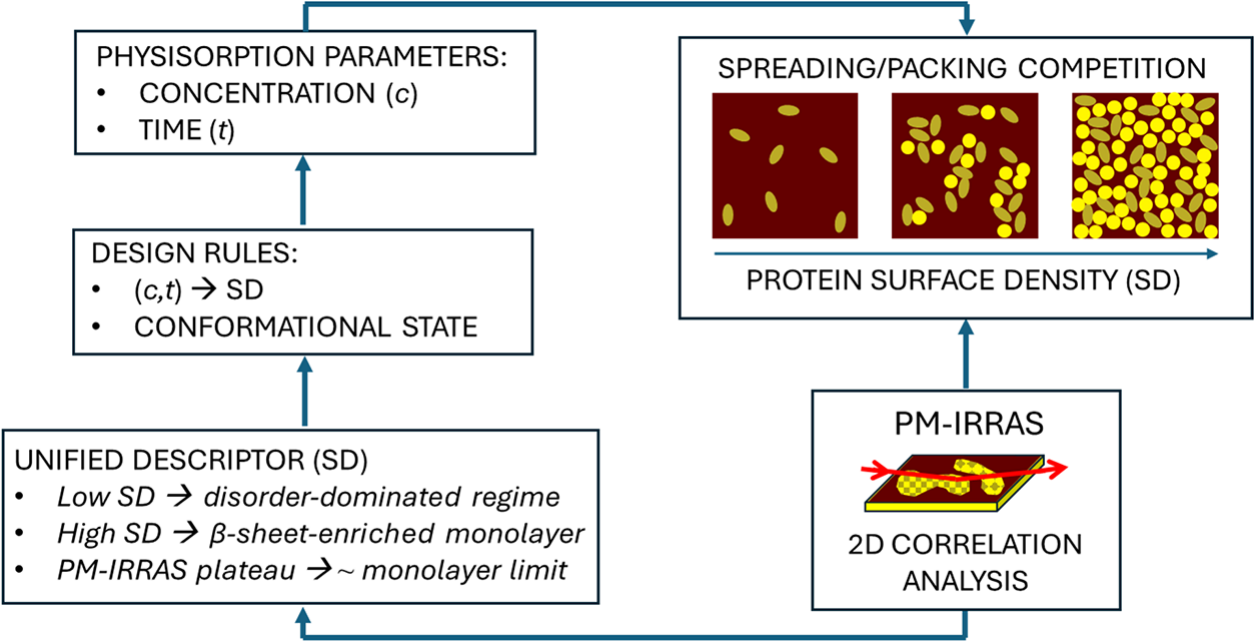
Conceptual block diagram illustrating the spreading–packing competition as the driving mechanism of structural heterogeneity in antibody monolayers. Adsorption conditions, i.e., solution concentration and deposition time, control the surface density (SD) of the forming adlayer. PM-IRRAS and two-dimensional correlation analysis quantify β-sheet and unordered fractions, revealing a sequential evolution from a spreading-controlled regime to a packing-controlled regime. The SD metric provides a unified descriptor linking adsorption conditions to structural outcomes and guiding the design of stable, function-preserving protein adlayers.

We employ PM-IRRAS coupled with 2D correlation analysis to quantify how adsorption parameters shape the antibody secondary structure. We investigate the adsorption of anti-immunoglobulin M (anti-IgM) on gold across a broad experimental space. Solution concentrations ranging from 0.5 to 1000 μg mL^–1^ and deposition times from 1 to 330 min span the transition from dilute to densely packed layers. To enable quantitative comparison across conditions, we introduce a normalized surface-density parameter (SD) derived from PM-IRRAS intensity, providing a dimensionless scale for adlayer evolution independent of absolute signal magnitude. By collapsing concentration- and time-dependent adsorption pathways onto a single surface-density coordinate, we provide a quantitative state variable that governs the structural evolution of antibody monolayers.

Our analysis reveals a robust anticorrelation between the β-sheet and unordered chain components within the amide I region. At low SD, antibodies adopt relaxed, partially unfolded conformations, whereas increasing SD promotes the retention of compact, β-sheet-rich assemblies. By mapping secondary-structure fractions versus SD, we identify the transition from disorder-dominated films to heterogeneous layers, containing comparable content of β-sheet and unordered chains. These findings establish surface density as the key descriptor controlling the spreading–packing balance in antibody monolayers and provide a quantitative framework for predicting adsorption-induced structural configurations.

The implications of this study are 2-fold. First, it provides a mechanistic framework linking adsorption parameters to structural outcomes in the antibody films. Second, it delivers quantitative guidelines, in terms of concentration, time, and SD, for engineering robust, function-preserving antibody interfaces. Physisorbed immunoglobulins on gold constitute a model system for sensing interfaces in optical and bioelectronic immunosensors, which exhibit high sensitivity to affinity binding.^16–18^ Controlling antibody structure at solid interfaces is essential for enhancing the detection performance of these biosensing platforms.^19^ Furthermore, the concepts developed here may extend to other protein–substrate systems where interfacial restructuring and ordering determine the adlayer morphology and functionality, offering general principles for the rational design of functional biointerfaces in materials science and biotechnology.

## 2 Results and Discussion

### 2.1 Adsorption-Dependent Conformational States Revealed by PM-IRRAS

Figure 2a shows PM-IRRAS spectra of anti-IgM monolayers physisorbed on gold, recorded after 70 min incubation in 4-(2-hydroxyethyl)-1-piperazineethanesulfonic acid (HEPES) buffer solutions with antibody concentrations from 0.5 to 100 μg mL^–1^. The spectra exhibit the characteristic IR bands of proteins, namely, the amide I band at ∼1670 cm^–1^, associated with C=O stretching, and the amide II band at ∼1540 cm^–1^, assigned to the combined N–H bending and C–N stretching modes of the polypeptide backbone.

**Figure 2:**
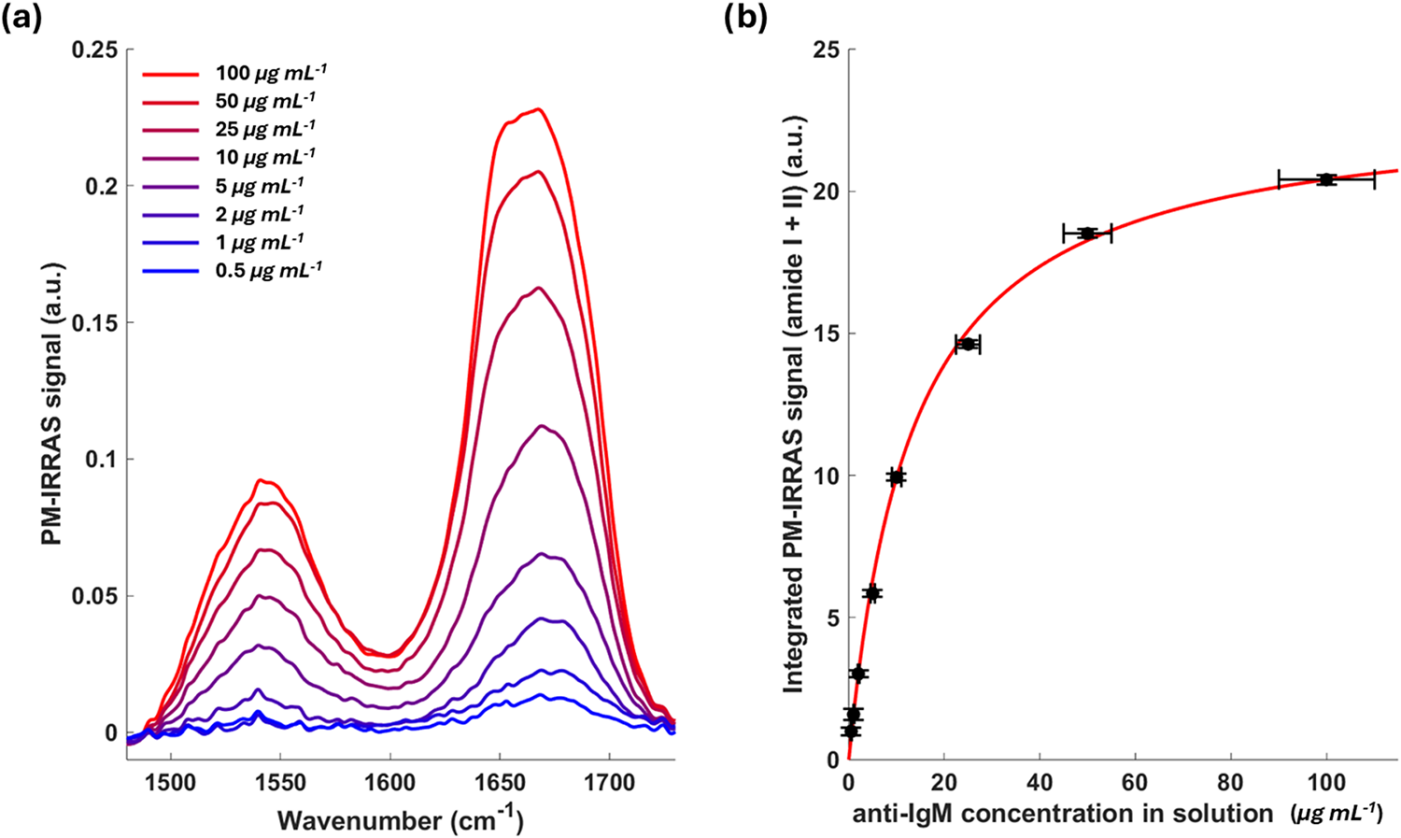
(a) PM-IRRAS spectra of human anti-IgM monolayers physisorbed onto gold surfaces after 70 min of deposition in HEPES buffer solution (pH = 7.4, i_s_ = 150 mM) at protein concentrations ranging from 0.5 to 100 μg mL^–1^, recorded in the amide I and II spectral regions. Each spectrum is baseline-corrected, and the background spectrum of a bare Au substrate was subtracted. (b) Integrated PM-IRRAS signal in the amide I–amide II range, 1510–1710 cm^–1^, (black dots) as a function of the anti-IgM concentration in solution. Y-error bars are the standard deviation measured over three replicate experiments; x-error bars are the uncertainty in concentrations computed by error propagation from the dilution procedure. The red line is a guide for the eyes. Measurements were repeated three times, showing an analogous trend to the reported representative one.

In Figure 2b, the integrated PM-IRRAS intensity under the amide I and amide II bands is plotted against the anti-IgM concentration of the bulk solution (*c*). The signal increases monotonically with the amount of adsorbed anti-IgM, while the plateau indicates that adsorption asymptotically approaches a maximum packing density in the monolayer limit within the explored concentration ranges. These results agree with recent surface plasmon resonance (SPR) reports.^18^

Moreover, although the trend in Figure 2b resembles a Langmuir-type profile, this model does not rigorously apply to soft-matter adlayers such as protein ones, due to their structural flexibility and solvent coupling.^9,20^ Once adsorbed, antibodies are expected to reorient and relax into metastable states of reduced free energy.^21^ The relaxation may involve partial unfolding and results in a spreading mechanism that maximizes protein–surface contact sites. Therefore, desorption becomes highly unlikely, making adsorption effectively irreversible.^8,9^ This is also supported by the in situ monitoring of anti-IgM adsorption and desorption kinetics on gold surface by Surface Plasmon Resonance (SPR) (Figure S1).

At low concentrations, the first anti-IgM molecules adsorb onto the surface and undergo reorientation and spreading. As adsorption proceeds, the available area decreases and steric hindrance limits further spreading, leading to progressively more compact packing up to complete surface coverage. Increasing the protein concentration in the solution accelerates surface filling, and the number of adsorbed molecules increases until apparent saturation of the available surface is reached to form a near-complete molecular monolayer.

### 2.2 Sequential Adsorption–Adaptation Mechanism Probed by 2D Correlation Analysis

PM-IRRAS is highly sensitive to protein orientation and to the local spatial conformation of the polypeptide backbone, i.e., the secondary structure.^13^ The amide I band contains information on the secondary structure main components: α-helices, β-sheets, turns, and unordered chains.^22,23^ To resolve the spectral variation associated with individual secondary structural motifs, we employ the 2D correlation analysis. The advantages of 2D correlation analysis are 2-fold: (i) it provides sharper and better resolved peaks than the original spectra and hence facilitates the identification of overlapping spectral components; (ii) it is ideal to highlight the individual and relative response of spectral features to changes in *c*.^14^ The method calculates cross-correlation maps that separate in-phase (synchronous) and out-of-phase (asynchronous) variations of the spectral intensities with respect to *c*.

The synchronous 2D contour map (Figure 3a) visualizes correlations between intensity variations at wavenumbers ν_1_, ν_2_ for the spectra of Figure 2a. Large autocorrelation peaks along the diagonal (ν_1_ = ν_2_) indicate the overall susceptibility of the spectral intensity to change in *c*. The autocorrelation function ϕ­(ν_1_ = ν_2_) plotted in the topmost panel of Figure 3a shows two intense bands in the ranges 1640–1690 and 1510–1570 cm^–1^ corresponding to the amide I and amide II vibrations, respectively. Particularly, the amide I band reveals a complex structure. The peak at 1647 cm^–1^ and the shoulder at 1691 cm^–1^ are due to B2 and B1 vibrational modes of β-sheets, respectively, while the feature at 1670 cm^–1^ is due to the unordered components. The off-diagonal cross-peak at (ν_1_ = 1665, ν_2_ = 1540) indicates that amide I and amide II vibrational bands increase together with *c*.

**Figure 3:**
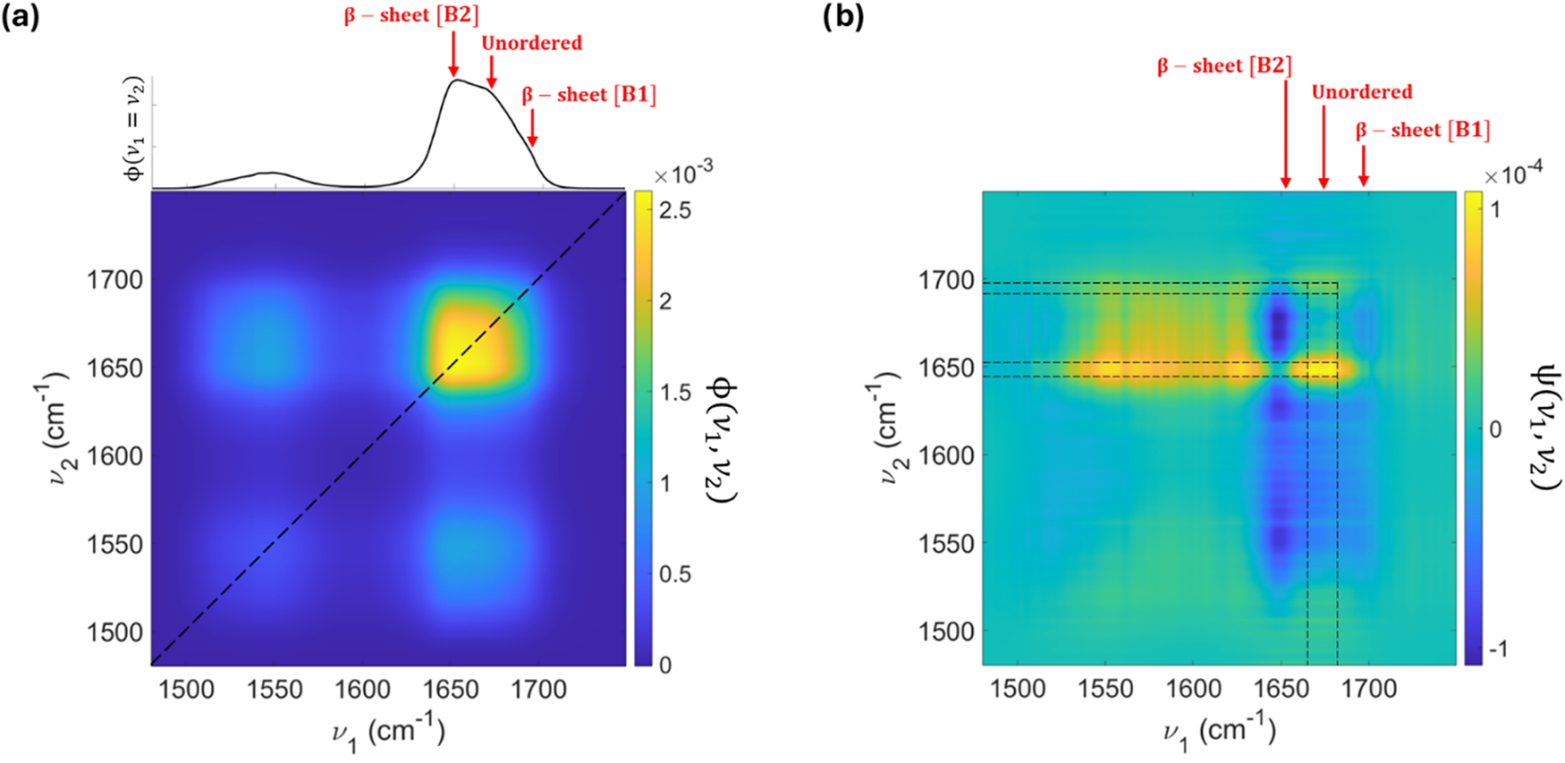
Synchronous (a) and asynchronous (b) 2D-COS map of PM-IRRAS spectra of human anti-IgM physisorbed on gold. Anti-IgM concentrations, varying in the range of 0.5–100 μg mL^–1^, are considered as a perturbation parameter for the analysis. The autocorrelation function ϕ­(ν_1_ = ν_2_) is reported on top of the synchronous map. The red arrows identify the secondary structure contributions to the amide I band.

Figure 3b shows the asynchronous 2D correlation map obtained from the spectra of Figure 2a. While the synchronous map identifies bands that vary together, the asynchronous map highlights features that evolve in different sequences or respond at different stages to the applied perturbation, i.e., the increase in *c*. Each cross-peak (ν_1_, ν_2_) of the asynchronous map represents two bands whose intensity variations are not simultaneous. The sign of the cross-peak indicates which band responds first to the external perturbation: the sign is positive if the absorption change induced by the increase in *c* at ν_1_ occurs before (i.e., at lower *c* values) that at ν_2_.^14^

The positive cross-peaks centered at (ν_1_, ν_2_)≡ (1670, 1647) cm^–1^ and (ν_1_, ν_2_)≡ (1670, 1691) cm^–1^ in Figure 3b indicate that at low *c* values, the main change occurs for the unordered components, whereas at higher concentration, the change of β-sheets prevails. Also, the negative region at (1640 <ν_1_ < 1690, 1530 <ν_2_ < 1600) reveals that spectral changes in the amide II band precede those in the amide I band. This observation indicates a sequential evolution of spectral components with increasing concentration. While this behavior is consistent with an early-stage modification of the adsorbed layer followed by subsequent changes in amide I contributions, the specific assignment of these processes to backbone reorientation and secondary-structure reorganization remains inferential. The same analysis was performed independently on three replicate data sets, yielding consistent synchronous and asynchronous features, confirming the reproducibility of the observed spectral trends.

The 2D correlation analysis thus reveals a progressive redistribution of secondary-structure content toward β-sheet-rich conformations.

### 2.3 Spectral Fitting

To quantitatively assess the secondary-structure composition of the antibody monolayer, the amide I band was deconvoluted using a four-Gaussian model (see the Experimental Section). The components correspond to (i) side-chains (∼1620 cm^–1^); (ii) β-sheets (B2, ∼ 1648 cm^–1^); (iii) unordered chains and turns (∼1671 cm^–1^); and (iv) β-sheets (B1, ∼1690 cm^–1^).^22,24^ The unordered contribution may include minor α-helical components due to spectral overlap in the 1660–1680 cm^–1^ region. However, given the low α-helical content of immunoglobulins, this does not affect the interpretation of relative trends.^24^

Figure 4a**–**c displays representative amide I fits for monolayers formed at 1, 10, and 100 μg mL^–1^, while Figure 4d and Table 1 summarize the relative fractions of the four structural components across the explored concentration range (1–100 μg mL^–1^). It should be noted that, in PM-IRRAS on metallic substrates, the intensity of vibrational bands depends not only on the population of structural motifs but also on the orientation of their transition dipole moments with respect to the surface. Therefore, changes in the relative amplitudes of fitted components may reflect a convolution of orientation and conformational effects.

**Figure 4:**
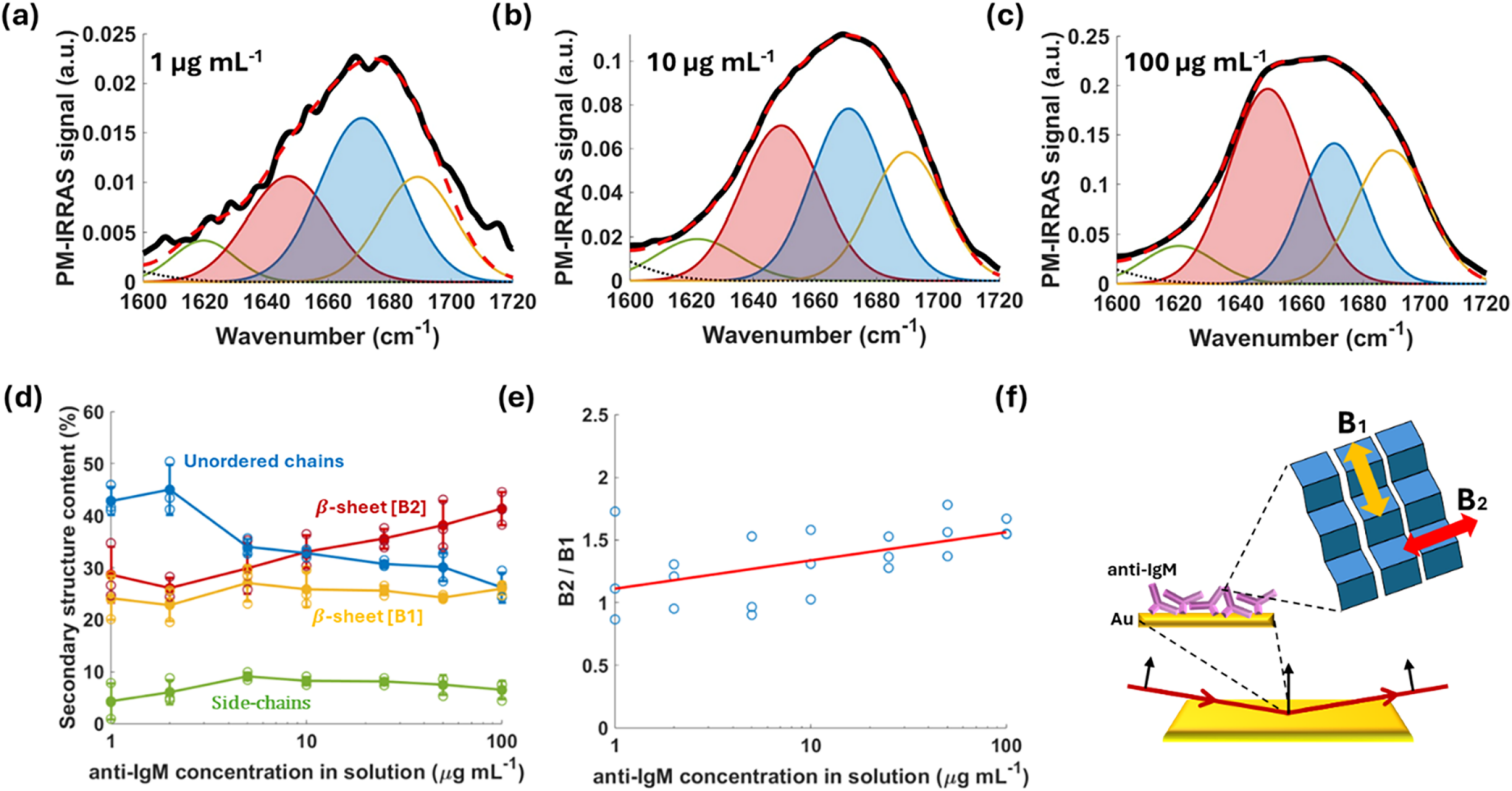
Fit-deconvolution and orientational analysis of the amide I band of PM-IRRAS spectra of human anti-IgM monolayers physisorbed on gold. (a–c) Representative amide I regions of spectra obtained after deposition from anti-IgM solutions at 1, 10, and 100 μg mL^–1^, respectively. (d) Relative fractions of secondary-structure components obtained from four-Gaussian deconvolution as a function of concentration. Filled circular markers and error bars correspond to the mean ± standard deviation of three replicate experiments. The hollow circles are the results of the individual experiments. (e) B2/B1 intensity ratio as a function of concentration. The red line is a linear fit to the data. (f) Schematic illustration of the grazing-incidence PM-IRRAS geometry and orientational sensitivity of β-sheet vibrations. The p-polarized beam detects dipole oscillations with a component normal to the gold surface (black arrow). Within each β-sheet, B1 (red) and B2 (green) denote perpendicular C = O transition dipoles lying along and across the β-strand direction, respectively. Increasing B2 relative to B1 indicates a larger tilt of β-strands toward the surface normal, consistent with adsorption-induced reorientation and compaction of β-domains.

**Table 1:** Secondary Structure Content of Anti-IgM Monolayer Obtained Following 70 min Deposition at Increasing Protein Concentration^a^

	secondary structure content (%)			
*c* (μg mL^–1^)	side-chain	β-sheet (B2)	unordered chains	β-sheet (B1)
1	4 ± 3	29 ± 5	43 ± 3	24 ± 4
2	6 ± 2	26 ± 2	45 ± 5	23 ± 3
5	9 ± 1	30 ± 5	34 ± 2	27 ± 3
10	8 ± 1	33 ± 3	33 ± 1	26 ± 3
25	8 ± 1	36 ± 2	31 ± 1	26 ± 1
50	7 ± 2	38 ± 5	30 ± 3	24 ± 0.4
100	6 ± 2	41 ± 3	26 ± 3	26 ± 1

a The reported secondary-structure fractions are the mean ± standard deviation over three replicate experiments

Results in Figure 4d show that the most significant variations involve the unordered and β-sheet (B2) contributions. The unordered fraction decreases monotonically from (43 ± 3)% to (26 ± 3)% as concentration increases, whereas the β-sheet (B2) fraction rises from (29 ± 5)% to (41 ± 3)%. This reciprocal evolution is consistent with a spreading-to-packing transition, in which increasing surface coverage progressively limits molecular relaxation and favors more compact β-structured conformations. Given the orientational sensitivity of PM-IRRAS, a combined orientational contribution cannot be fully excluded.

Figure 4e features a monotonic increase in the *B*2/*B*1 ratio of ∼40%. Because of the orientational sensitivity of PM-IRRAS, changes in the *B*2/*B*1 intensity ratio reflect changes in the average β-sheet orientation. The β-sheet features centered at ≈ 1648 cm^–1^ (B2) and ≈ 1690 cm^–1^ (B1) originate from vibrational coupling within the same β-sheet motifs, corresponding to perpendicular C=O transition dipoles and their transition dipoles are along and perpendicular to the β-strand direction, respectively, as schematically illustrated in Figure 4f.^25^ Therefore, an increase of B2 relative to B1 indicates that β-strands acquire a larger tilt toward the surface normal (more upright orientation).

Concomitantly with increasing β-sheet fraction, the decrease in unordered intensity evidences a redistribution of spectral weight from flexible to β-structured regions. This coupled evolution reflects adsorption-driven orientational ordering and compaction within pre-existing β-sheets. Notably, the unordered contribution arises from a distribution of randomly oriented dipoles and is therefore expected to be less sensitive to preferential orientation effects and more directly reflect unordered chain abundance. The result is consistent with previous PM-IRRAS studies of antibody monolayers on gold and with recent evidence of orientation-dependent antibody organization at Au interfaces.^13,26^

Additional fitting tests, including extra components (e.g., α-helix/turn contributions), showed that the extracted β-sheet fractions remain stable, confirming the robustness of the four-component model.

### 2.4 Deposition Time Dependence

To assess the impact of adsorption time on the structural composition of the antibody adlayer, anti-IgM monolayers were formed on gold using deposition times of 1 and 330 min, at protein concentrations *c* = 5, 50, 500, and 1000 μg mL^–1^.

Figure 5 compares representative amide I PM-IRRAS spectra for *c* = 5 μ g mL^–1^ and 1000 μg mL^–1^after short (1 min) and long (330 min) deposition times. At low concentration (5 μg mL^–1^, Figure 5a,b), short exposure produces an adlayer dominated by unordered chains (≈ 50%) and a minor β-sheet (B2) fraction (≈ 27%). Prolonged deposition promotes structural rearrangement, yielding a 19% increase in β-sheet content and a corresponding reduction in disordered components (to ≈ 43% unordered chains, 32% β-sheet).

**Figure 5:**
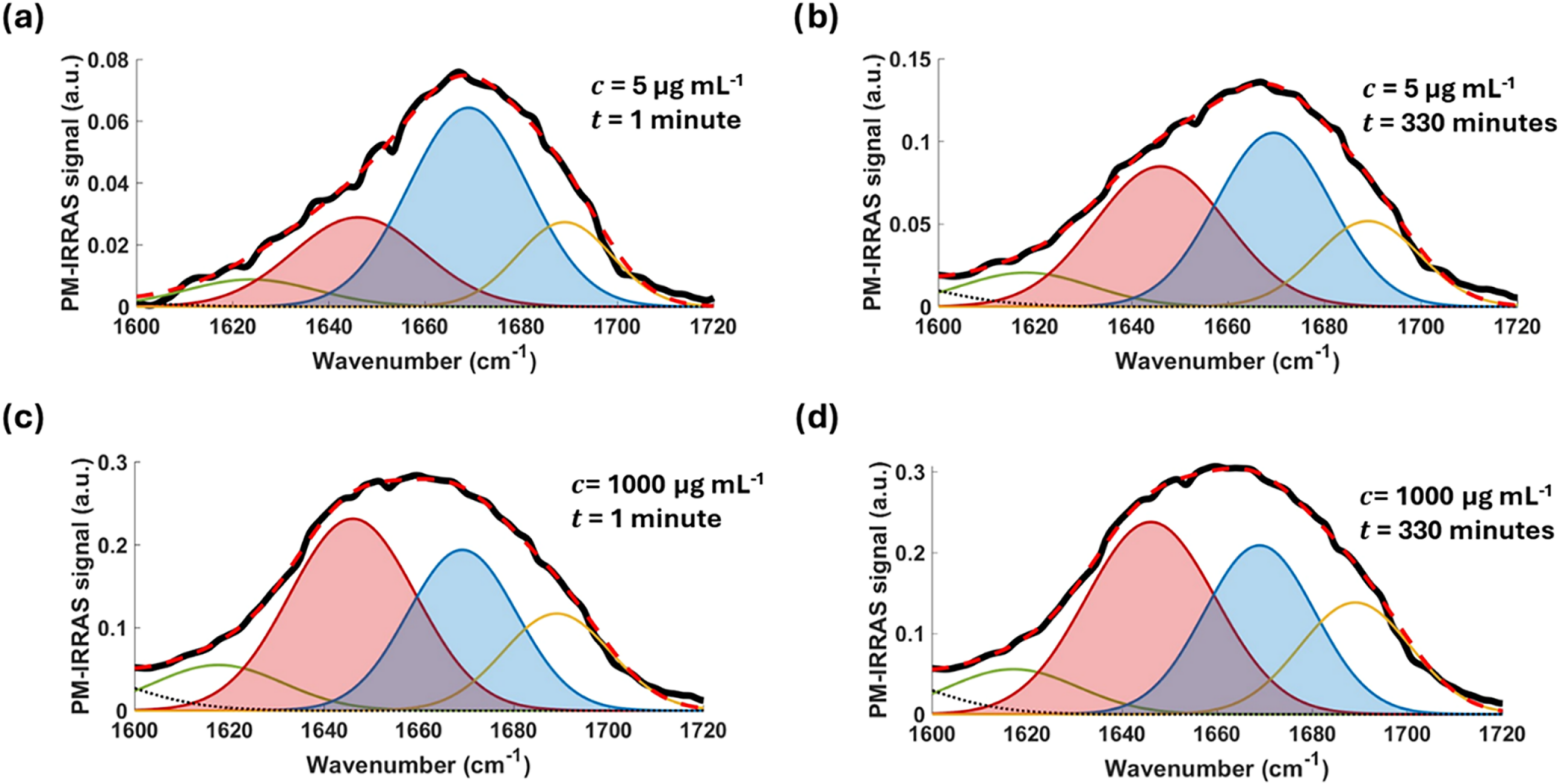
Time dependence of secondary-structure composition in anti-IgM monolayers on gold. PM-IRRAS amide I spectra and corresponding Gaussian deconvolution for different deposition times (t) and protein concentrations (c): *t* = 1 min and c = 5 μg mL^–1^ (a), *t* = 330 min, c = 5 μg mL^–1^ (b), *t* = 1 min, c = 1000 μg mL^–1^ (c), *t* = 330 min, c = 1000 μg mL^–1^ (d). The main sub-bands are assigned to β-sheet (B2, red) and unordered (blue) components. Low-concentration adsorption allows conformational adaptation over time, while high-concentration deposition yields rapid surface saturation and time-independent structure.

In contrast, at the highest concentration (1000 μg mL^–1^, Figure 5c,d), the secondary-structure composition remains nearly unchanged over time, with both unordered and β-sheet fractions ≈ 35%. Intermediate concentrations (50–500 μg mL^–1^; Table 2) exhibit a gradual transition between these two regimes.

**Table 2:** Secondary Structure Content of Anti-IgM Monolayer Obtained Following Deposition at Various Times (t) and Protein Concentrations (c)^a^

		secondary structure content (%)			
t (min)	*c* (μg mL^–1^)	side-chain	β-sheet (B2)	unordered chains	β-sheet (B1)
1	5	6 ± 1	27 ± 0.4	50 ± 0.3	17 ± 0.4
1	50	8 ± 3	33 ± 3	43 ± 6	17 ± 2
1	500	9 ± 1	34 ± 2	39 ± 1	17 ± 1
1	1000	9 ± 1	36 ± 1	35 ± 3	19 ± 1
330	5	10 ± 1	33 ± 1	41 ± 2	16 ± 1
330	50	10 ± 2	35 ± 1	38 ± 1	17 ± 2
330	500	10 ± 1	37 ± 2	35 ± 1	18 ± 1
330	1000	10 ± 1	36 ± 2	34 ± 1	20 ± 0.4

a The reported secondary-structure fractions are the mean ± standard deviation over three replicate experiments

These results confirm that the adsorption process evolves from a time-dependent regime, in which molecules can reorient and restructure on the surface, to a coverage-limited regime, where rapid surface saturation and steric constraints suppress the conformational adaptation. The observed time independence at high concentration indicates that the antibody layer attains near-complete coverage within the first minute, after which further adsorption or restructuring is sterically hindered.

The persistence of concentration-dependent differences even after 330 min deposition (see Figure 5 and Table 2) demonstrates that protein–protein lateral interactions are insufficient to restore a native-like β-sheet content and supports the notion that the films retain an inherent heterogeneity. In other words, even in a near-complete monolayer, a mixed population of proteins with different levels of unfolding exists.

Further insight into the time dependence of anti-IgM physisorption was provided by in situ monitoring of adsorption kinetics by attenuated total reflection infrared spectroscopy (ATR, Figure S2). The ATR data reveal an initial diffusion-limited adsorption regime, followed by a surface-coverage-limited phase leading to saturation within 330 min. Notably, the observed plateaus correspond to ∼100% surface coverage, while different saturation levels indicate higher protein packing as *c* increases. This is further supported by Kelvin probe force microscopy (KPFM) nanoscale mapping (Figure S3) showing that a ∼86% coverage is reached after just 90 s at 50 μ*g mL* ^–1^.

### 2.5 Normalized Surface Density as a Unifying Descriptor

To quantitatively unify the influence of concentration and deposition time, we introduce a normalized surface-density (SD) parameter derived from PM-IRRAS integrated intensity. Rather than treating concentration and time as independent variables, SD represents a structural state coordinate that reflects the molecular packing in a near-complete monolayer coverage. In this framework, adsorption pathways characterized by different kinetic histories can be projected onto a single dimensionless axis governing structural evolution. The maximum integrated intensity (*I*_max_) of the amide I band, obtained for a 330 min deposition at 1000 μg mL^–1^, is taken as the reference corresponding to maximum packing. The SD is then expressed as SD = *I*/ *I*_max_ where I is the integrated intensity of the amide I band for a given spectrum. All spectra are thus expressed as a fraction of the maximum value, providing a dimensionless scale of relative surface occupancy.

Figure 6a**–**c shows representative amide I spectra and their deconvolution for SD = 19%, 51%, and 93%. At low SD (≈19%), the spectra are dominated by unordered components, indicating the prevalence of spread, partially relaxed antibodies. As SD increases, β-sheet features strengthen while unordered contributions decline, consistent with the progressive restriction of molecular spreading under packing constraints. Figure 6d,6e features the secondary-structure evolution and the B2/B1 intensity ratio as a function of SD. Bovine anti-IgM was utilized in this case, so while the observed trends are consistent with those reported for human anti-IgM in Figure 4d,e, variations could be ascribed to species-dependent differences.

**Figure 6:**
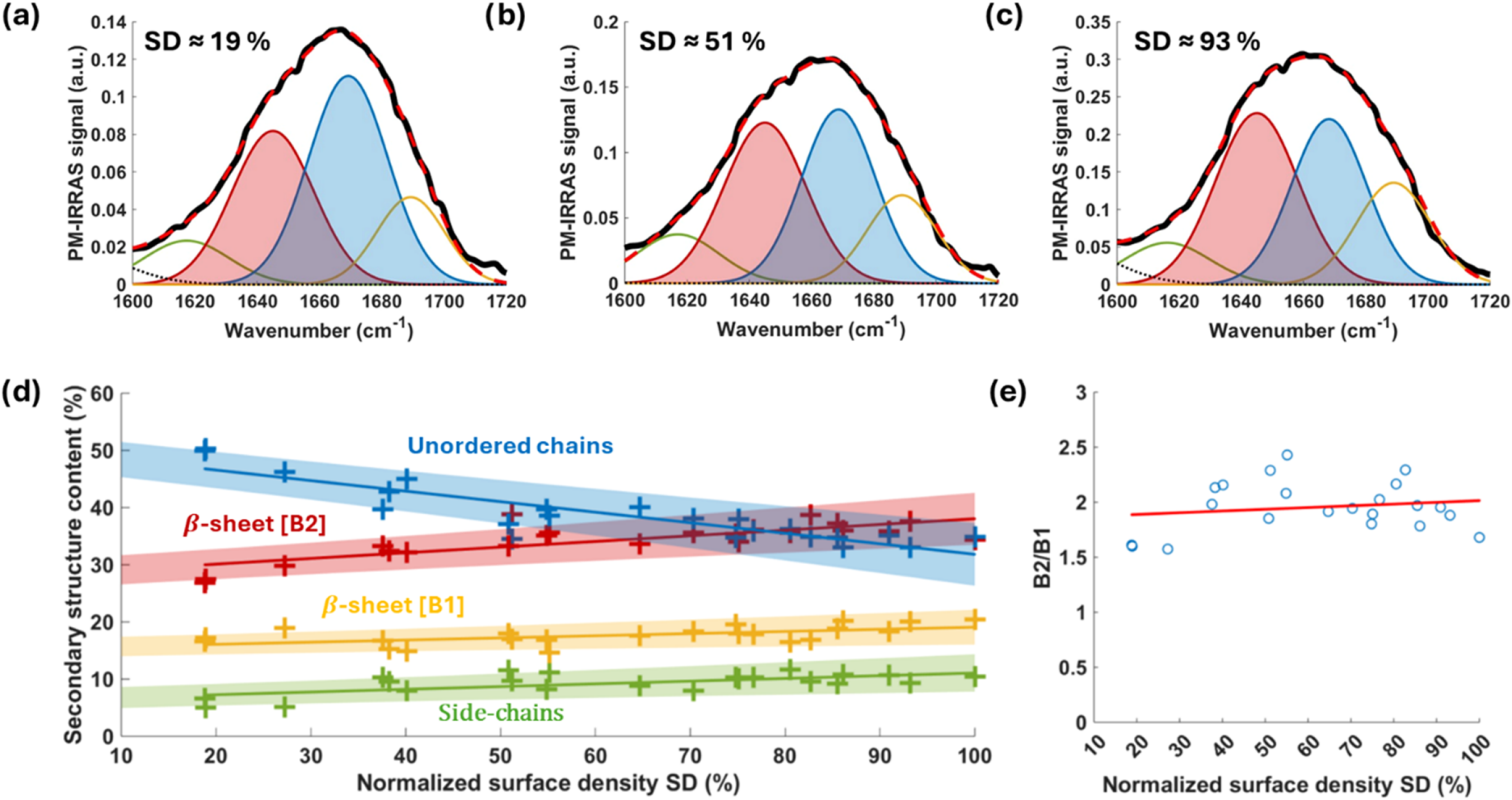
Secondary structure content vs normalized surface-density (SD) of anti-IgM monolayers on gold. (a–c) Amide I PM-IRRAS spectra and Gaussian deconvolution for SD = 19%, 51%, and 93%, corresponding to progressively denser adlayers. (d) Evolution of secondary-structure fractions as a function of SD. Symbols represent individual experimental data points, while shaded areas indicate 95% confidence intervals of the linear fits. PM-IRRAS intensities are normalized to the maximum value obtained for a 330 min deposition at 1000 μg mL^–1^, corresponding to near-complete monolayer coverage. (e) B2/B1 intensity ratio as a function of SD. The red line is a linear fit to the data.

Within this framework, SD represents the physically meaningful variable governing the conformational evolution of the adlayer. It should be considered that the PM-IRRAS intensity depends on both surface coverage and molecular orientation due to polarization selection rules. Therefore, SD reflects a convolution of these effects and should be interpreted as a relative descriptor of the spectroscopically active interfacial density rather than as an absolute quantity.

SD enables the quantitative comparison of specific protein–substrate interfaces obtained under different adsorption pathways (i.e., varying *c* and *t*). Compared with surface density derived from other mass-sensitive techniques such as SPR or quartz crystal microbalance, the spectroscopically defined SD is a comparative descriptor sensitive to the conformational heterogeneity of the adlayer.

Classical spreading–packing models of protein adsorption describe competition between surface-induced unfolding and lateral crowding effects. However, these models are typically formulated qualitatively and lack a direct spectroscopic mapping onto experimentally measurable structural fractions. Here, by combining PM-IRRAS with 2D correlation analysis and expressing structural evolution as a function of normalized surface density, we provide a quantitative spectroscopic realization of this competition for antibody monolayers on metallic surfaces.

## 3 Conclusion

In summary, we demonstrate that antibody monolayer structure is governed by a surface-density-dependent competition between spreading and packing. By introducing a normalized surface-density parameter and combining it with 2D correlation spectroscopy, we identify trends consistent with a sequential adsorption–reorientation–restructuring pathway and quantitatively map the evolution of β-sheet and unordered spectral contributions. At low surface coverage, molecular spreading dominates, allowing extensive protein–surface interaction and partial unfolding that generates conformational disorder. As surface density increases, intermolecular packing imposes steric constraints that favor retention of native-like structural features, i.e., β-sheet. The coexistence of these distinct conformational populations reflects the balance between spreading forces—promoting extended, partially unfolded states—and packing constraints that stabilize compact β-sheet-rich assemblies. Notably, our data provides no evidence of refolding driven by lateral intermolecular interactions.

Although demonstrated here for anti-IgM on gold, the methodological approach, linking adsorption pathways to structural state variables, may be extended to other proteins and substrates in which adsorption-induced structural adaptation plays a functional role. For example, structurally soft proteins, like immunoglobulins or albumin, on hydrophobic substrates, are prone to adsorption-induced conformational changes that are better captured as a function of SD than surface coverage.

Conversely, the applicability of the SD framework may be limited in cases where proteins are small and structurally rigid, with minimal conformational adaptation upon adsorption, or adsorption is fully reversible and well-described by equilibrium isotherms.

This proposed framework provides a quantitative descriptor for predicting how solution concentration and deposition time determine the interfacial structure of physisorbed antibody monolayers. By tuning these parameters, or equivalently, the SD metric, specific structural states can be selectively targeted to balance the structural stability and biological functionality. Reliable modulation of interfacial conformation is essential for engineering antibody coatings that retain activity and robustness, directly impacting the sensitivity, reproducibility, and lifetime of biosensing, plasmonic, and bioelectronic devices.

## 4 Experimental Section

### 4.1 Materials

Antihuman and antibovine Immunoglobulin M (anti-IgM, Sigma-Aldrich) were used as received, buffered in aqueous solution at ∼2 mg mL^–1^ and ∼46 mg mL^–1^, respectively. A 4-(2-hydroxyethyl)-1 piperazine-ethane-sulfonic acid (HEPES, MW ≈ 238.30 g mol^–1^) buffered solution (pH 7.4, ionic strength is = 150 mm) was utilized to dilute proteins at the desired final concentration. It was prepared by dissolving the HEPES dry powder in Milli-Q water (∼18 MΩ cm) to obtain 200 mL of 5 mM HEPES solution. The pH was adjusted to 7.4 by adding ∼0.4 mL of 1 M sodium hydroxide (NaOH Fisher Scientific). The ionic strength was adjusted by adding 1.67 g of sodium chloride (NaCl, MW ≈ 58.44 g mol^–1^, BioXtra, ≥ 99.5%, ATMerck) dry powder.

### 4.2 Substrate Cleaning and Functionalization

PM-IRRAS reflective metallic substrates were prepared by deposition of a Ti/Au (5/50 nm) metallic film onto glass microscope slides, by electron-beam evaporation. The glass slides were previously cleaned via sequential ultrasonic bath in water, acetone, and 2-propanol, for 10 min. For the substrate functionalization, the gold surfaces were treated in a UV-ozone cleaner for 10 min and incubated with 1 mL of anti-IgM solution for the desired deposition time (1, 70, or 330 min) at a certain concentration of proteins (in the range from 0.5 to 1000 μg mL^–1^). Afterward, the functionalized substrates were immersed in an aqueous solution of HEPES 5 mM to remove excess deposited salts and dried via spinning for 3 min at 1500 rpm.

### 4.3 Polarization-Modulation Infrared Reflection–Absorption (PM-IRRAS) Spectroscopy

PM-IRRAS spectroscopy measurements were performed by a Thermo Scientific apparatus, including a Nicolet iS50 FTIR spectrometer coupled to an external research module. The setup includes a ZnSe photoelastic modulator (PEM), a sample holder, a liquid nitrogen-cooled mercury–cadmium–telluride (MCT) detector, and a synchronous sampling demodulator electronic system. The PEM modulates the polarization of the incident light beam, periodically switching it from the s- to p-state (corresponding to the electric field oscillating parallel and perpendicular to the sample surface) at 100 kHz. The sample is illuminated at a grazing incidence angle (82°). Acquired PM-IRRAS spectra are averaged over 1000 scans at 4 cm^–1^ resolution, with PEM modulation efficiency peaked at 1670 cm^–1^. All spectra are baseline-corrected, using a second-order polynomial baseline that was computed by the least-squares method^27^ in the range of 1480–1800 cm^–1^, and the spectrum of bare Au was subtracted before further analysis.

### 4.4 Two-Dimensional (2D) Correlation Analysis of PM-IRRAS Spectra

The PM-IRRAS spectra of Figure 2a, measured as a function of the external perturbation variable *c*, are subjected to cross-correlation calculations to generate 2D contour maps.^14^ In these maps the *x*- and *y*-axes are independent wavenumber axes (ν_1_, ν_2_). Preliminarily, the set of spectra recorded with unevenly spaced increments of the external variable was converted into evenly spaced data through interpolation.^14^ So-called dynamic spectra are computed as *Y*_*i*_(ν) = *y*_*i*_(ν) – *y̅*(ν), where *y*_*i*_(ν) is the i-th PM-IRRAS spectrum and *y̅*(ν) = ∑_*i* = 1_^*n*^ *y*_*i*_(ν) is the reference spectrum taken as the average spectrum, as usual. Here, *n* is the total number of spectra.

The synchronous 2D correlation intensity map is computed from the dynamic spectra as $\varphi \left(\nu_{1},\nu_{2}\right)=\frac{1}{n-1}\sum_{i=1}^{n}Y_{i}\left(\nu_{1}\right)Y_{i}\left(\nu_{2}\right)$ . The asynchronous 2D correlation intensity map is calculated as $\psi \left(\nu_{1},\nu_{2}\right)=\frac{1}{n-1}\sum_{i=1}^{n}Y_{i}\left(\nu_{1}\right)\sum_{k=1}^{n}N_{\mathrm{ik}}Y_{k}\left(\nu_{2}\right)$ where *N*_*ik*_ is the Noda-Hilbert transform matrix, defined as $N_{\mathrm{ik}}=\left\{ \begin{array}{ll} 0, & \mathrm{if}\;i=k\\ 1/\pi \left(k-i\right), & \mathrm{otherwise} \end{array}\right.$ .^14^ The calculations are performed using a custom MATLAB code.

### 4.5 Amide I Fit-Deconvolution

The fit-deconvolution of the amide I bands was performed by a custom MATLAB code, in the spectral range (1610–1695) cm^–1^. The fit model includes five Gaussian functions, corresponding to four secondary structure contributions, plus an additional one accounting for the high-energy tail of the amide II band protruding beyond 1610 cm^–1^. The number of components under the amide I band is defined based on the second-derivative spectra (see Figure S4). To ensure robustness of the deconvolution and limit overfitting, peak positions were constrained within narrow ranges based on literature assignments and second-derivative analysis, while bandwidths were allowed to vary within physically reasonable limits (22–32 cm^–1^). Initial parameter values were systematically varied, and consistent trends in the relative contributions of spectral components were obtained across the data set, confirming that the fitting results are not sensitive to the specific choice of initial conditions or constraint ranges. Intensities, bandwidths, and positions of individual Gaussian curves simultaneously and independently adjust to fit experimental data. The resulting fit-curve exhibits a root mean squared error less than 0.9 × 10^–3^ and an adjusted R-squared larger than 0.97 (see Table S1). Representative residual plots are reported in Figure S5. The four sub-bands were assigned to side-chains (∼1620 cm^–1^), β-sheets (∼1647 cm^–1^ and ∼1690 cm^–1^), and unordered chains (∼1671 cm^–1^).^13^

## Supplementary Material



## Notes

In-situ monitoring of anti-IgM adsorption kinetics by Surface Plasmon Resonance (Notes: S1); in-situ monitoring of anti-IgM adsorption kinetics by Attenuated Total Reflectance Spectroscopy (Notes: S2); nanoscale surface coverage assessment by Kelvin probe Force Microscopy (KPFM) (Notes: S3); and second-derivative analysis S5: Amide I fit-deconvolution: Four-Gaussian model (Notes: S4) (PDF)

## #

Institute of Photonic Sciences (ICFO), Barcelona, Spain
