# Supplementary material for: Surface-Density-Controlled Spreading–Packing Competition in Antibody Monolayers Revealed by PM-IRRAS and 2D Correlation Spectroscopy
Source: ACS Omega. 2026 Jun 17;11(25):37430–9. doi: 10.1021/acsomega.6c02134 (PMC13325138; doi:10.1021/acsomega.6c02134)
Supplement: Supplementary file 1 [file ao6c02134_si_001.pdf]

Supporting Information

Surface-Density–Controlled Spreading–Packing Competition in Antibody Monolayers Revealed by PM-IRRAS and 2D Correlation Spectroscopy

Matteo Piscitelli[1,2], Cinzia Di Franco [2], Lucia Sarcina [3], Michele Catacchio [4], Fabrizio Corvino[1], Eleonora Macchia [4], Luisa Torsi [3], and Gaetano Scamarcio[5]**

[1] Dipartimento Interateneo di Fisica, Università degli Studi di Bari Aldo Moro, Bari 70125, Italy

[2] Consiglio Nazionale delle Ricerche – Istituto di Fotonica e Nanotecnologie (CNR-IFN), Bari 70125, Italy

[3] Dipartimento di Chimica, Università degli Studi di Bari Aldo Moro, Bari 70125, Italy

[4] Dipartimento di Farmacia-Scienze del Farmaco, Università degli Studi di Bari Aldo Moro, Bari 70125, Italy

[5] Consiglio Nazionale delle Ricerche – Istituto Nanoscienze (CNR-Nano), Pisa 56127, Italy

S1. In-situ monitoring of anti-IgM adsorption kinetics by Surface Plasmon Resonance

Multi-Parameter Surface Plasmon Resonance (MP-SPR) in the Kretschmann prism coupling configuration is used to monitor the adsorption kinetics of anti-IgM immunoglobulins on gold. The commercial system NaviTM 200 by BioNavis, equipped with two 670 nm lasers, included a 0.1 mL flow-cell hosting a 0.5 cm^2 glass slide coated with Cu/Au. The SPR slide surface was exposed to protein solutions through static injection mode, at concentration equal 100 µg mL^{-1}, 50 µg mL^{-1}, 25 µg mL^{-1}, 10 µg mL^{-1}, in HEPES buffer (is = 150 mM; pH 7.4). Figure S1 features the SPR sensogram, acquired over hours-long time. The sensograms show similar trend to those measured by ATR spectroscopy, showing a fast initial physisorption kinetics followed by a slower one. In order to explore the desorption kinetics as well, the measuring cell was rinsed with plain HEPES buffer. The blue arrows indicate the rinsing of the cell. Notably, the SPR signal after rinsing shows no significant desorption, demonstrating that protein adsorption is irreversible over an hour-long period.

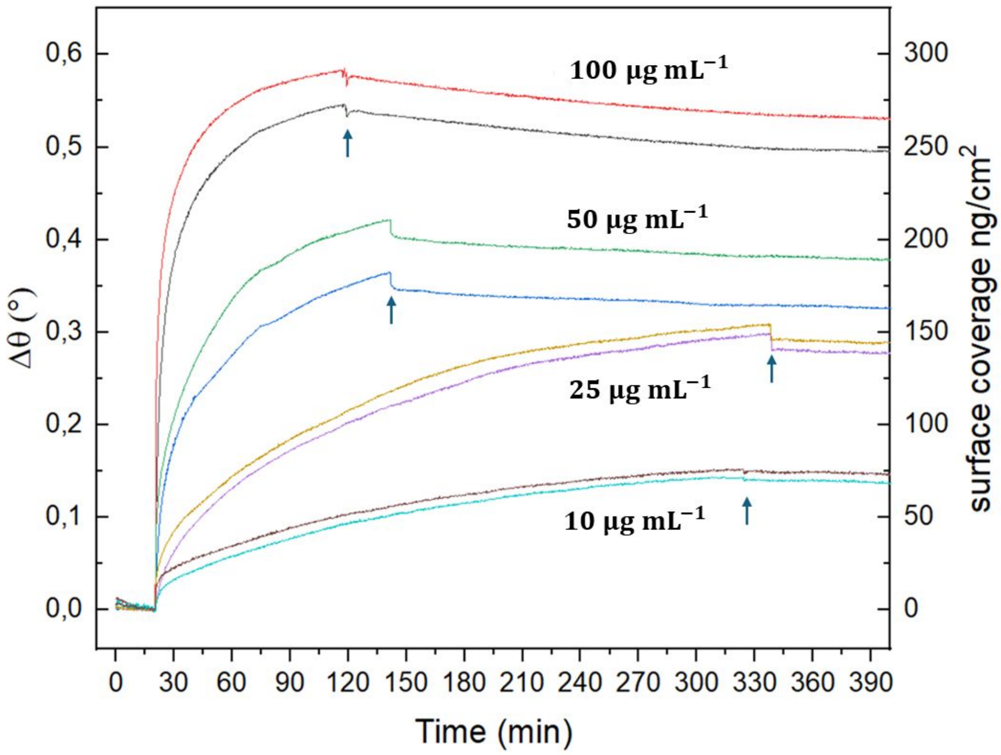

Figure S1. SPR sensograms of anti-IgM immunoglobulins adsorbed onto the gold from HEPES buffer solution of proteins at bulk concentration equal 100 μg mL^{-1} 50 μg mL^{-1} (yellow), 25 μg mL^{-1}, 10 μg mL^{-1}. Two sensograms are recorded during each measurement corresponding to the two lasers illuminating the SPR plate. The blue arrows indicate the rinsing of the measuring cell with plain HEPES buffer. The surface coverage is evaluated as described elsewhere.[1]

S2. In-situ monitoring of anti-IgM adsorption kinetics by Attenuated Total Reflectance Spectroscopy

Attenuated total reflectance (ATR) spectroscopy was employed to monitor the adsorption kinetics of anti-IgM immunoglobulins. The ATR spectra of adsorbed anti-IgM were collected

using a Nicolet iS50 FTIR spectrometer (Thermo Scientific) equipped with a Smart iTX accessory featuring a single-bounce diamond prism illuminated at a 45° incidence angle.

A background spectrum of the plain HEPES buffer (I = 150 mM; pH 7.4) was first recorded. Subsequently, 50 µL of the anti-IgM solution was deposited onto the diamond surface, and automated acquisition of consecutive spectra was initiated. The sample was covered with a glass lid to minimize solvent evaporation during the measurements. Spectra were recorded over a 5-hour period with a time resolution of approximately 1 minute, each averaged over 16 scans. All measurements were carried out at room temperature. The spectra were corrected for atmospheric water vapor and carbon dioxide interference and background-subtracted in the amide region. The integrated ATR absorbance in the 1500–1700 cm^{-1} range, encompassing the amide I and amide II bands, was evaluated and used as a quantity proportional to the amount of protein adsorbed on the diamond surface. **Figure S2** shows the time evolution of the signal for three protein concentrations: 50, 500, and 1000 µg mL^{-1}. The signal exhibits a monotonic increase, with an initial rapid rise followed by a slower approach to saturation, consistent with the adsorption kinetics discussed in the main text.

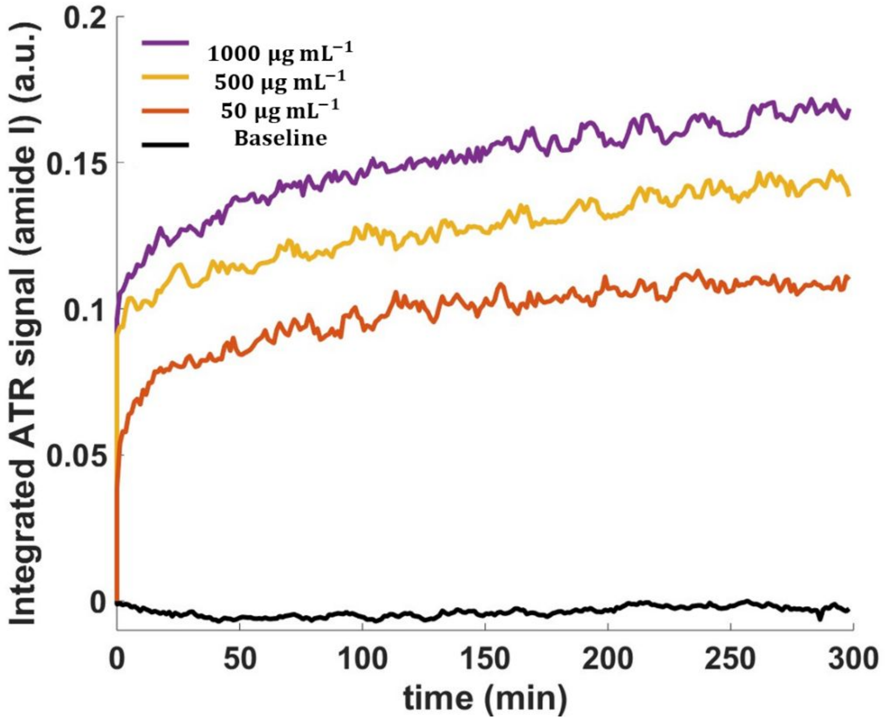

Figure S2. ATR absorbance of anti-IgM immunoglobulins adsorbed onto the diamond surface. The lines represent the integrated ATR absorbance in the amide I – amide II wavenumber range, measured in-situ upon protein deposition from solution at bulk concentration of 1000 µg mL^{-1} (purple) 500 µg mL^{-1} (yellow), 50 µg mL^{-1} (orange). The black line corresponds to the integrated ATR absorbance measured with the bare diamond surface.

S3. Nanoscale surface coverage assessment by Kelvin probe Force Microscopy (KPFM)

Single-pass sideband KPFM measurements were carried out using a Park NX10 atomic force microscope. Imaging was performed over 500 × 500 nm^2 regions with a spatial resolution of 1 nm per pixel. Conductive cantilevers supplied by Park Systems for KPFM applications (Multi 75M, spring constant 2 N/m, resonance frequency 62 kHz, Pt/Ir coating) were used. The

amplitude and frequency of the modulation voltage were 1 V and 3 kHz, respectively. The tip–sample distance was controlled via a setpoint of 10 nm, and the scan rate was maintained at 0.2 Hz per line. Lock-in amplifier parameters were optimized to ensure high signal-to-noise ratio under these conditions.

Si/SiO_2 (300 nm) substrates were used. They were cleaned in an ultrasonic bath (10 min each) in water, acetone, and isopropanol (all the solvents were VLSI grade). Anti-IgM biofunctionalized samples were obtained via physisorption for 90 s, at room temperature, followed by rinsing in HPLC-grade water and drying by spin-coating (3000 rpm, 60 s). Samples were prepared using three concentration incubation solutions: 0.5, 5, and 50 µg mL^{-1} in HEPES buffer (150 mM ionic strength, pH 7.4).

Figure S3 shows AFM morphologies and KPFM surface-potential maps of anti-IgM monolayers on SiO_2 prepared using an incubation time of 90 s and anti-IgM concentrations of 0.5, 5, and 50 µg mL^{-1}. At 0.5 µg mL^{-1} (**Figure S3a**), adsorption is sparse: isolated protrusions assigned to single anti-IgM molecules exhibit heights of ~2 nm and apparent lateral widths of ~15 nm (broadened by tip–sample convolution), consistent with a flat-on configuration. At 5 µg mL^{-1} (**Figure S3c**), coverage increases, and neighboring islands develop necked contacts, indicating incipient coalescence and partial percolation while island boundaries remain well defined. At 50 µg mL^{-1} (**Figure S3e**), an almost packed granular layer forms with a reduced void fraction and segment-like motifs.

We report the KPFM signal as the surface potential SP, defined as

$$SP = \frac{W_{tip} - W_S}{e} \qquad (1)$$

where W_{tip} and W_S are the work functions of the inspecting tip and sample respectively and e the elementary charge. In general, the work function is described as $W = W^{Bulk} + \phi$, including

the sum of a bulk contribution (W^{Bulk}) and a surface one (ϕ). Using the surface potential measured on bare SiO_2 as the reference, we define the surface-potential difference (SPD) as:

$$SPD = SP_{anti-IgM} - SP_{SiO_2} = \frac{\left(\Phi_{SiO_2} - \Phi_{anti-IgM}\right)}{e} \qquad (2)$$

The KPFM maps mirror the coverage-dependent evolution seen in the topography. At 0.5 µg mL^{-1} (**Figure S3b**), the maps show a uniform SiO_2 background punctuated by isolated bright spots that correspond to the topographic protrusions. These features indicate localized molecular dipoles associated with single anti-IgM adsorbates and yield a potential offset of ~80 mV with respect to the substrate. At 5 µg mL^{-1} (**Figure S3d**), the surface-potential maps display domains whose contrast closely follows the morphological features; the SPD is ~100 mV. Thin rims and small step-like offsets at inter-island boundaries point to lateral variations in dipole density and/or orientation during coalescence. At 50 µg mL^{-1} (**Figure S3f**), the potential distribution becomes substantially more homogeneous, as expected for a quasi-continuous dipole sheet. As the film evolves from isolated domains to a near-continuous layer, the laterally connected dipoles blankets and electrostatically screens the substrate. Consequently, contrast differences between the anti-IgM layer and bare SiO_2 are progressively less visible, and the SPD cannot be determined, at the highest coverage.

To quantify the areal coverage, grain analysis was performed on both the height and surface potential channels, following plane subtraction and denoising procedures.

A binary mask of candidate protrusions was generated by simultaneously applying height and surface potential thresholds, with respect to the local background. The surface coverage was calculated as the percentage area fraction (surface coverage) covered by the mask. The orange regions are anti-IgM-covered areas as identified by the binary mask. At 0.5 µg mL^{-1}, the areal coverage is ~5% of the imaged field (**Figures S3 a-b**); at 5 µg mL^{-1} (panels c-d), coverage

increases to ≈ 26%. At 50 μg mL⁻¹ (**Figures S3 e–f**), a granular layer covers ~86% of the surface.

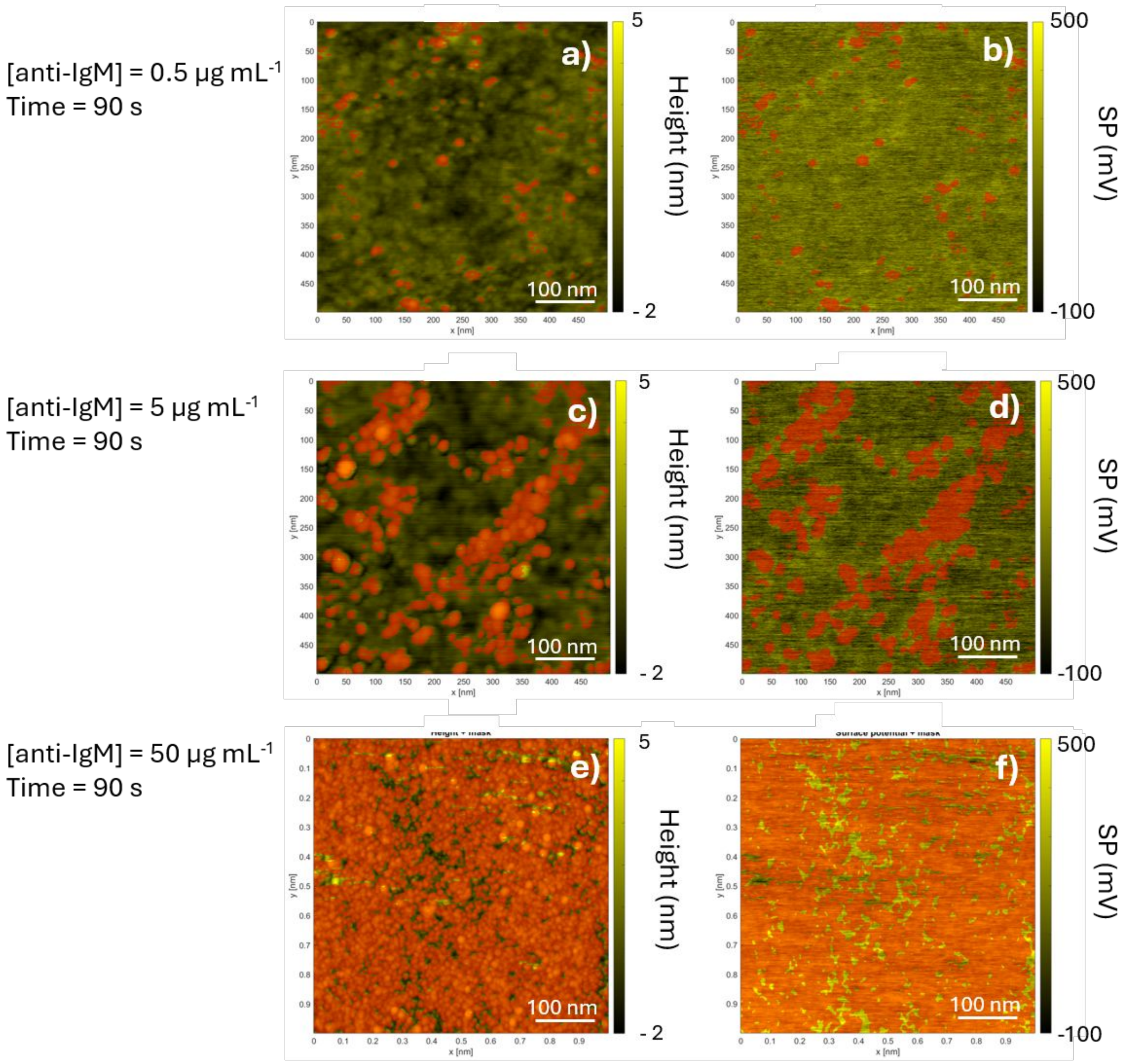

Figure S3. Morphology and surface potential (SP) images of Si/SiO_2 sample surfaces, modified with physisorbed anti-IgM following a 90-second incubation at concentration of 0.5 μg mL^{-1}(**a-b**) 5 μg mL^{-1}(**c-d**), and 50 μg mL^{-1} (**e-f**). Height and SP values are relative the Si/SiO_2 background, taken as zero level.

S4. Second-derivative analysis

The second derivative of the PM-IRRAS spectra of physisorbed anti-IgM at concentrations ranging from 1 μg mL⁻¹ to 100 μg mL⁻¹ was calculated using a Savitzky–Golay filter that fits a third-order polynomial to a sliding window 11.5 cm⁻¹ wide, to determine the number and positions of secondary structural components within the amide I region. **Figure S4** shows representative second-derivative spectra of anti-IgM samples obtained upon physisorption from bulk solutions with increasing protein concentrations c = 2, 10, and 100 μg mL⁻¹. Minima in the second-derivative spectra indicate the presence of individual spectral components underlying the amide I band.

As shown in **Figure S4b**, two distinct minima are observed at 1648 cm⁻¹ and 1690 cm⁻¹, associated to β-sheets components which are expected to be predominantly present in Immunoglobulins. To account for the second-derivative line shapes in the regions between 1610–1630 cm⁻¹ and 1660–1680 cm⁻¹, at least two additional components must be considered, even though they do not produce sharp minima due to the overlap with adjacent components. Notably, comparison of the second-derivative spectra in **Figure S4** reveals that at high concentrations the band at 1648 cm⁻¹ dominates, whereas its relative contribution decreases as the concentration is reduced.

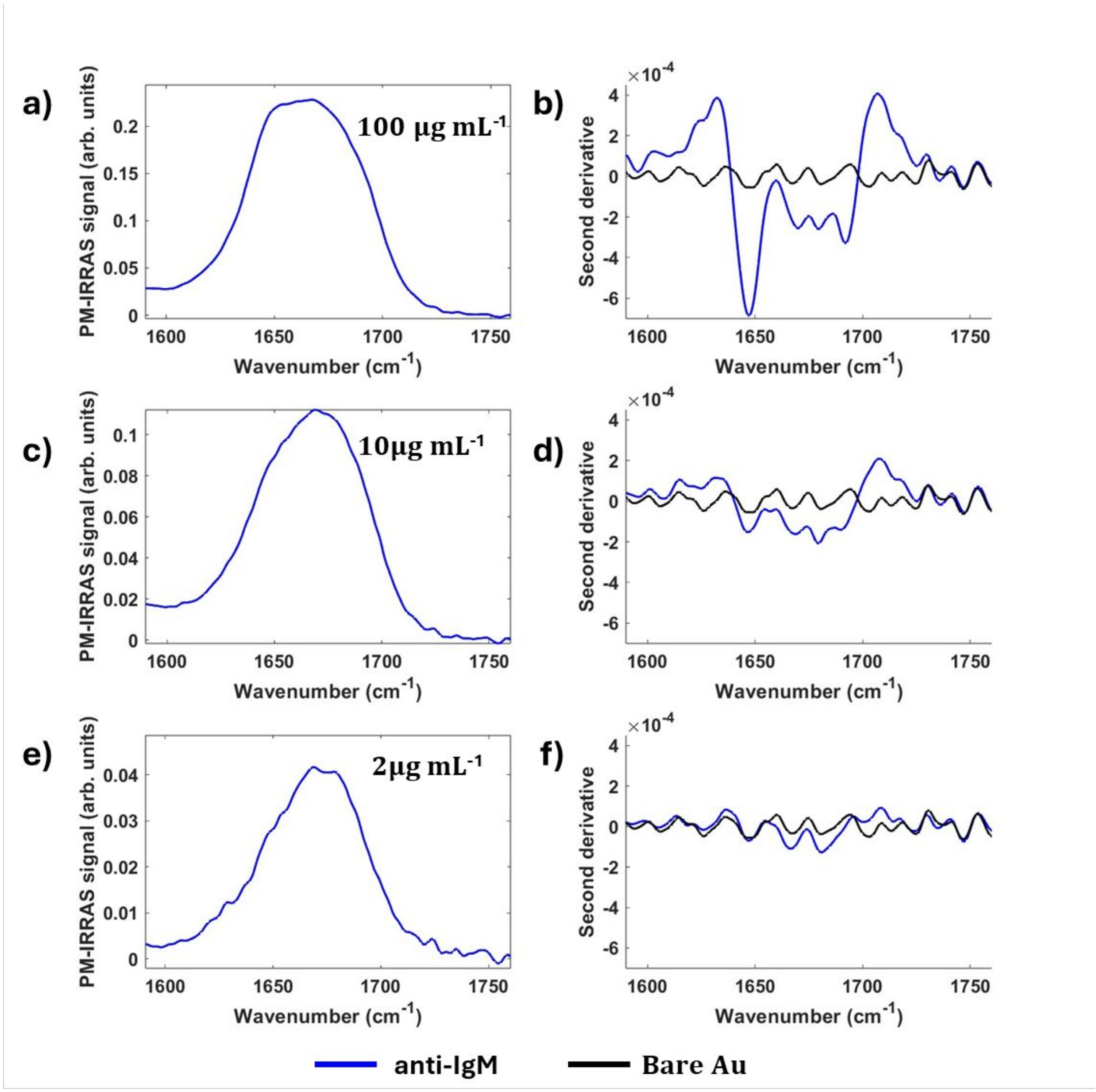

Figure S4. PM-IRRAS spectrum and its second derivative of human anti-IgM monolayers on gold, obtained via deposition at 2 µg mL^{-1} (a-b) 10 µg mL^{-1} (c-d) and 100 µg mL^{-1} (e-f). The second derivative spectrum of a bare gold substrate is also shown (black line) as a reference baseline.

S5. Amide I fit-deconvolution: Four-Gaussian model

The performance of the four-Gaussian model was evaluated through the root mean squared error (RMSE) and adjusted R-squared (R^2) metrics. Figure S5 shows the fit results of for the representative spectra shown in Figure 2a of the main text, along with the corresponding residual plots. Notably, residual plots show only minor structured features, which can be attributed to residual water vapor absorption lines that are not fully cancelled by polarization modulation in PM-IRRAS measurements. No systematic deviations related to the amide I band are observed, confirming that the four-Gaussian model adequately reproduces the experimental lineshape. Table S1 reports the resulting RMSE and R^2.

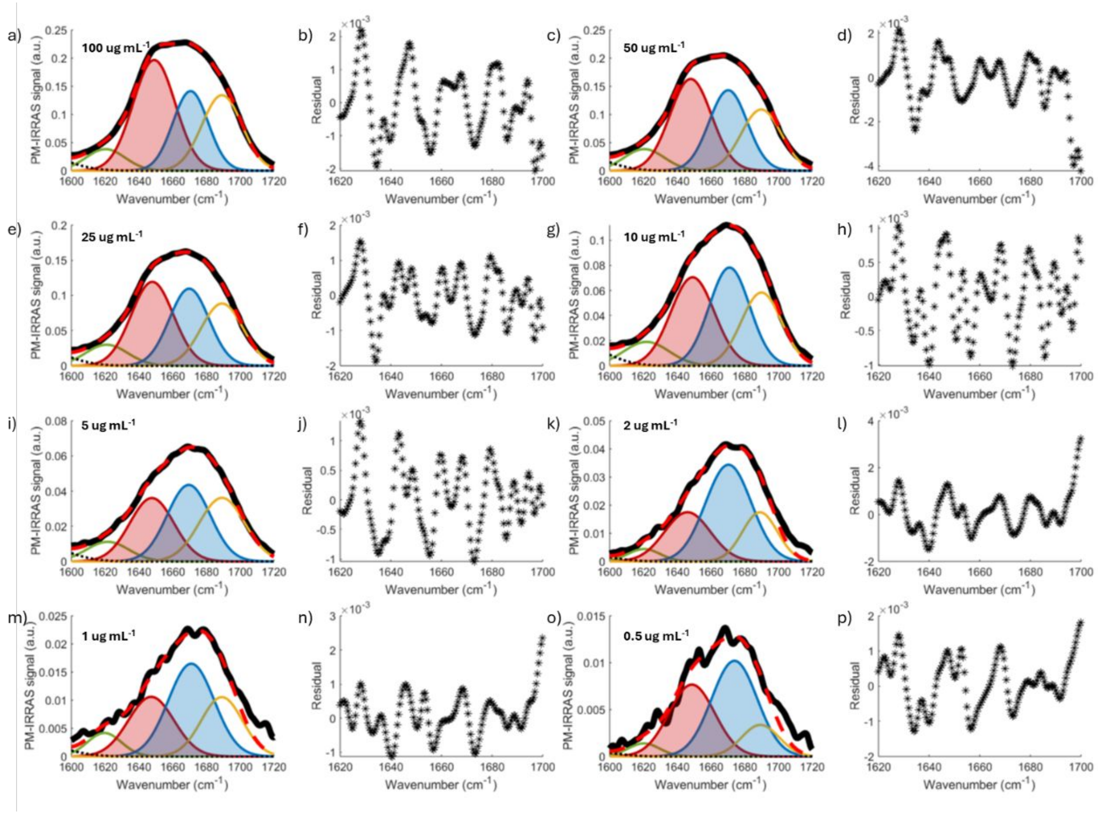

Figure S5. Fit-deconvolution of the amide I band of PM-IRRAS spectra of human anti-IgM monolayers physisorbed on gold (a,c,e,g,i,k,m,o) along with the corresponding residual plots (b,d,f,h,j,l,n,p) obtained after deposition from anti-IgM solutions at 0.5, 1, 2, 5, 10, 25, 50, and 100 µg mL^{-1}, respectively.

Concentration (µg mL^{-1})	R^2	RMSE (10^{-3})
100	0.9998	0.9
50	0.9998	0.9
25	0.9998	0.7
10	0.9997	0.5
5	0.9991	0.6
2	0.9978	0.6
1	0.9940	0.5
0.5	0.9752	0.6

Table S1. R-squared (R^2) and Root mean squared error (RMSE) corresponding to the fit reported in figure S5.

References

[1] L. Sarcina, C. Scandurra, C. Di Franco, M. Caputo, M. Catacchio, P. Bollella, G. Scamarcio, E. Macchia and L. Torsi, A stable physisorbed layer of packed capture antibodies for high-performance sensing applications, J. Mater. Chem. C, 2023,11, 9093-9106
